# Developmental Impacts of Early Sensory Patterns on School-Age Adaptive, Maladaptive, and Participation Outcomes in Autistic and Non-autistic Children

**DOI:** 10.1007/s10803-024-06494-0

**Published:** 2024-08-02

**Authors:** Yun-Ju Chen, John Sideris, Linda R. Watson, Elizabeth R. Crais, Grace T. Baranek

**Affiliations:** 1 Mrs. T. H. Chan Division of Occupational Science and Occupational Therapy, University of Southern California, 1540 Alcazar Street CHP 133, Los Angeles, CA 90089 USA; 2 Program for Early Autism Research, Leadership and Service (PEARLS), University of North Carolina at Chapel Hill, Chapel Hill, NC USA; 3 Department of Occupational Therapy and Graduate Institute of Behavioral Sciences, College of Medicine, Chang Gung University, Taoyuan, Taiwan; 4 Department of Health SciencesDivision of Speech and Hearing Sciences, Department of Health Sciences, University of North Carolina at Chapel Hill, Chapel Hill, NC USA

**Keywords:** Autism, Sensory patterns, Developmental trajectories, School-age outcomes, Longitudinal structural equation modeling

## Abstract

Early sensory differences may cascade into later social-communication difficulties in autism, yet their impacts on broader functional outcomes have remained understudied. This study aimed to conduct a comprehensive investigation into the longitudinal impacts of sensory patterns, including sensory hyperresponsiveness, hyporesponsiveness, and sensory repetitions/seeking behavior, on various school-age outcome domains among a community sample of children with autistic and non-autistic conditions. We prospectively followed 1,517 children with caregiver-reported sensory questionnaires across three timepoints from infancy to school age. A subsample (n = 389; 88 with reported autism diagnosis/concerns) was further assessed with adaptive, maladaptive and participation outcome measures at age 6–7. Structural equation modeling approaches were used to evaluate the multivariate associations between latent growth parameters (i.e., intercepts and slopes) of sensory patterns and school-age outcomes. Increasing sensory hyperresponsiveness was directly associated with poorer adaptive/maladaptive outcomes and indirectly with lower participation in activities with higher functional demands across settings at school age. Elevated sensory hyporesponsiveness was associated with lower adaptive functioning, more externalizing problems, and lower classroom participation. Trajectories of sensory patterns accounted for more unique variances in adaptive functioning and participation in daily life settings with higher functional and environmental demands among autistic children compared to their non-autistic peers.

**Supplementary Information:**

The online version contains supplementary material available at 10.1007/s10803-024-06494-0.

## Introduction

Sensory patterns, including hyperresponsiveness (HYPER; e.g., distressed when touched), hyporesponsiveness (HYPO; e.g., tuning out sounds), and sensory interests, repetitions, and seeking behaviors (SIRS; e.g., fascinated with lights), are commonly observed in autistic children, with prevalence rates ranging from 74 to 94% (Baranek et al., 2006; Ben-Sasson et al., 2019; Kirby et al., 2022). The incorporation of sensory patterns into the restricted, repetitive behaviors criteria in the DSM-5 (DSM-5; American Psychiatric Association, 2013), along with longitudinal evidence from population-based and infant-sibling studies indicating their early emergence and association with later autism outcomes (Chen et al., 2022b, 2024; Riboldi et al., 2023; Wolff et al., 2019), reflect the crucial role of sensory patterns in diagnosing and supporting autistic children. Beyond the autism spectrum, the estimated prevalence of elevated sensory patterns in population-based samples of school-aged children was 5 to 8% (Ahn et al., 2004; Jusilla et al., 2019). As sensory patterns are present across the neurodevelopmental continuum, it is important to understand their impact on other behavioral domains, such as adaptive functioning and maladaptive behavior. This knowledge can aid in identification and intervention efforts for both autistic and non-autistic children with sensory differences.

Previous literature has documented the associations between sensory differences and social communication and daily living skills (Feldman et al., 2020; Liss et al., 2006; Watson et al., 2011; Williams et al., 2018), motor skills (Jasmin et al., 2009; Surgent et al., 2021; Tomchek et al., 2015), emotional-behavioral problems (Baker et al., 2008; Dellapiazza et al., 2020; Green et al., 2012), and daily activity participation (Hochhauser & Engel-Yeger, 2010; Little et al., 2015; Reynolds et al., 2011) across autistic and non-autistic populations. However, the findings were mixed due to the methodological differences in study designs, purposes, measures, and participant characteristics across studies. For instance, while previous research has generally indicated a negative association between sensory differences and adaptive functioning (Feldman et al., 2020; Liss et al., 2006), other studies have reported null findings (McCormick et al., 2016; O’Donnell et al., 2012) or differential associations for specific subdomains or subgroups (Dellapiazza et al., 2020; Kirby et al., 2019; Tomchek et al., 2015; Watson et al., 2011; Williams et al., 2018). The varied findings may be attributed to the heterogeneous manifestations of sensory differences across the neurodevelopmental spectrum, coupled with their potential variations over time or across contexts. Previous studies may have been limited in addressing these complexities when relying on observed total scores without considering potential multidimensionality and measurement errors for capturing sensory patterns, alongside conventional analytical methods that may not adequately address individual variability (Uljarević et al., 2017).

Furthermore, it is worth noting that the existing evidence on sensory patterns is largely based on cross-sectional data from age-heterogeneous groups, which limits the ability to draw developmentally relevant implications. Longitudinal evidence is essential to support the potential cascading impacts of sensory patterns on later developmental outcomes (Baranek et al., 2018; Thye et al., 2018) as demonstrated by emerging evidence from prospective infant studies (Grzadzinski et al., 2020; Worthley et al., 2023). For instance, a toddler who exhibits hyperresponsiveness to certain sensory experiences may miss out on opportunities to explore their environment, learn new skills, or interact with others. This, in turn, could lead to difficulties in higher-order functioning later in life. Longitudinal evidence demonstrating these cascading effects is clinically significant, as it can inform early intervention strategies, including tailored environmental supports to optimize long-term behavioral outcomes for children with sensory differences (Baranek et al., 2018; Uljarević et al., 2017). Furthermore, the cascading impacts of sensory patterns on more distal or higher-level functional outcomes, such as daily activity participation, remain understudied and thus necessitate further investigation.

To address these empirical and methodological gaps, we applied structural equation modeling approaches, including latent growth curve modeling and path analysis, to investigate the multivariate associations between early development of sensory patterns and school-age adaptive/maladaptive and participation outcomes in a prospective birth cohort. Additionally, we sought to compare the impacts of sensory development in children with and without an autism condition to assess the relative significance of early sensory patterns on school-age outcome, including adaptive, maladaptive, and participation outcomes. Our hypothesis was that certain sensory patterns, characterized by higher initial levels and larger increases throughout early childhood, would be differentially associated with poorer school-age outcomes. We also hypothesized that the developmental trajectories of sensory patterns (hereafter “sensory trajectories”) would explain a greater amount of variance in school-age outcomes for autistic children compared to their non-autistic peers.

## Methods

### Participants and Procedure

This study involved a prospective birth cohort of 1,517 children born in 2013 recruited from the North Carolina birth registry. Families of Hispanic/Latino ethnicity were excluded from recruitment because the FYIv3.1 had not been translated into Spanish at the time of the study (see Chen et al., 2022b, 2024 for study details). The caregivers provided consent for their children to participate and were followed across three timepoints to report their child’s development on various domains of behavior using paper forms and/or online questionnaires when the child was aged 6–19 months (Time 1; T1), 3–4 years (Time 2; T2), and 6–7 years (Time 3; T3). Sensory patterns were assessed with the First Years Inventory version 3.1 (FYIv3.1; Baranek et al., 2013) at T1, and Sensory Experiences Questionnaire version 2.1 (SEQv2.1; Baranek et al., 1999) at T2 and T3-Phase 1. During the second phase of T3, approximately 5 months after the Phase-1 responses were collected, a subset of families (N = 465) was invited via email invitations to complete school-age outcome measures, including Vineland Adaptive Behavior Scales, Third Edition (VABS-3; Sparrow et al., 2016) and Participation and Environment Measure-Children and Youth (PEM-CY; Coster et al., 2010). This subset included families (N = 359) who reported any diagnosis/concerns via the (DCQv1.5; Reznick et al., 2005) and elevated autistic traits via the Social Responsiveness Scale, Second Edition (SRS-2; Constantino & Gruber, 2012). Additionally, a random sample of families whose responses did not indicate concerns at any previous time points was included (N = 106). A total of 389 responses were received across all the timepoints, with 312 (80%) of them reporting the presence of a developmental diagnosis and/or concerns at T2 or T3.

For the current study, the children with complete T3 outcome data (subsample; N = 389) were classified into either the autistic group (N = 88; reported by parents to have an autism diagnosis from clinicians via the DCQ and/or met the SRS-2 total T-score ≥ 60 cutoff at T2 or T3), or non-autistic group (N = 301; not meeting the autistic group criteria). Table 1 shows the demographic and clinical characteristics of the full sample (N = 1,517; with complete data of sensory measures) and the subsample (N = 389; with school-age outcome data) by autistic and non-autistic groups.

**Table 1:** Sample demographic and clinical characteristics

	Full sample (N = 1517)	Subset sample w/ school-age outcomes (N = 389)	
		Autistic (N = 88)	Non-autistic (N = 301)
Demographic characteristics			
Sex (male)	742 (49%)	65 (74%)	168 (43%)
Race			
White	1,315 (87%)	71 (81%)	270 (90%)
Black	65 (4%)	5 (6%)	6 (2%)
Asian	16 (1%)	1 (1%)	3 (1%)
American Indian/Hawaiian	11 (1%)	0 (0%)	4 (1%)
Multi-racial/other	110 (7%)	11 (12%)	18 (6%)
Parent education (5% missing)			
Both parents had a college degree (or beyond)	896 (59%)	33 (38%)	172 (57%)
One of the parents had a college degree (or beyond)	328 (22%)	24 (27%)	71 (24%)
None of the parents had a college degree (or beyond)	209 (14%)	27 (31%)	42 (14%)

Clinical characteristics by school-age			
Reported non-autistic developmental diagnosis or concerns	–	82 (93%)	187 (62%)

		Mean (SD)	
SRS-2 total T-score	–	69.66 (9.91)	48.07 (5.28)
VABS-3 domain-level standard scores			
Adaptive skills (composite)	–	84.12 (12.24)	101.28 (12.63)
Motor skills	–	91.17 (14.32)	102.39 (11.15)
Internalizing behavior	–	18.59 (2.54)	15.53 (2.76)
Externalizing behavior	–	18.41 (2.63)	15.34 (2.93)

### Measures

#### Sensory Patterns

Children's sensory patterns were assessed using two parent-report measures, both utilizing a 5-point Likert scale: the FYIv3.1, an early screener for identifying infants aged 6–16 months at elevated likelihood for a later diagnosis of autism (Baranek et al., 2013) and the SEQv2.1, a questionnaire for measuring the behavioral responses to daily sensory experiences for children ages 9 months through 12 years (Baranek et al., 1999). The FYIv3.1 has shown appropriate structural validity (Baranek et al., 2022) and has been applied in several studies involving community samples and high-risk samples (e.g., Meera et al., 2020; Turner-Brown et al., 2013). The SEQv2.1 has demonstrated appropriate structural validity (Lee et al., 2022), excellent internal consistency (alpha = 0.80) and test–retest reliability (ICC = 0.92) (Little et al., 2011), and has shown utility in differentiating autistic children from those with typical development or other developmental conditions (Baranek et al., 2006). It has been widely used in previous research on sensory differences in autism (e.g., Boyd et al., 2010; Wolff et al., 2019).

In the current study, comparable item-response-theory-based scores across measures and timepoints were computed using a common set of parent-reported items across the two measures for longitudinal analysis, with higher scores indicating more elevated sensory patterns (for details, see Appendix in the supplementary materials).

#### Clinical Outcome Classification

As described in the Participants and Procedure section, the DCQv1.5 and SRS-2 were used to classify participants into autistic and non-autistic groups. The DCQv1.5 is a parent-report measure with open-ended questions about whether a caregiver or professional (e.g., psychiatrists, pediatricians, or psychologists) had been concerned about the child’s development and whether the child has received any clinical diagnoses (Reznick et al., 2005). The DCQv1.5 has demonstrated utility in previous research on community samples for clinical outcome ascertainment (Chen et al., 2022b, 2024; Turner-Brown et al., 2013). The SRS-2 is a widely-used parent-report measure of autistic traits with established general-population norms (Constantino & Gruber, 2012). A total T-score (M = 50, SD = 10) ≥ 60 suggests clinically significant social difficulties and elevated likelihood of an autism diagnosis. Participants were classified into the autistic group if they were reported to have an autism diagnosis through the DCQ and/or those who met the SRS-2 T-score cutoff at T2 (3–4 years) or T3 (6–7 years).

#### Adaptive and Maladaptive Outcomes

The VABS-3 is a widely used standardized measure of adaptive functioning from birth to age 90. The domain-level parent/caregiver form was used to assess children’s adaptive skills, motor skills, and maladaptive behavior (two subscales: internalizing and externalizing behaviors). The standardized scores (M = 100, SD = 15 for the adaptive behavior composite and motor skills and M = 15, SD = 3 for maladaptive behavior subscales) were used for the current analysis.

#### Activity Participation Outcomes

The PEM-CY is a parent-report questionnaire designed to assess participation in 25 types of daily activities across three settings: home, school, and community. Caregivers reported on the child’s participation frequency (0 = never to 7 = daily) and extent of involvement (1 = minimally involved to 5 = very involved) for each activity. The internal consistency of the frequency scales ranged from insufficient to acceptable (α = 0.59-0.70), while the involvement scales showed acceptable to good consistency (α = 0.72-0.83) (Coster et al., 2011).

To capture both the frequency and level of involvement in operationalizing participation outcomes for the current study, a new variable was created by multiplying the item-level responses from the frequency and involvement scales, resulting in scores ranging from 0 to 35. These new scores served as indicators for common factor analysis, which was conducted using maximum-likelihood estimation and varimax factor rotation to determine the internal factor structures for each of the home, school, and community settings. The number of factors was determined using multiple criteria, such as scree plots with parallel analysis (eigenvalues > simulated values), relative fit indices (Tucker-Lewis Index [TLI] > 0.90 and root-mean-square error of approximation [RMSEA] < 0.06), and theoretical consistency.

As shown in Figure S1, three factors were extracted for the home setting, two factors for the school setting, and three factors for the community setting. Two community items (“*working for pay*” and “*overnight trips*”) were dropped due to limited relevance to the current young sample and low standardized factor loading (< 0.20) across the extracted factors, respectively. Bartlett’s approach was utilized to derive factor scores, which provide unbiased estimates of the true factor scores (Hershberger, 2005). These derived factor scores were then used as the distal outcomes in the primary analysis. Descriptive statistics of these factor scores by clinical outcome groups can be found in Table S1.

### Statistical Analyses

**Fig. 1:**
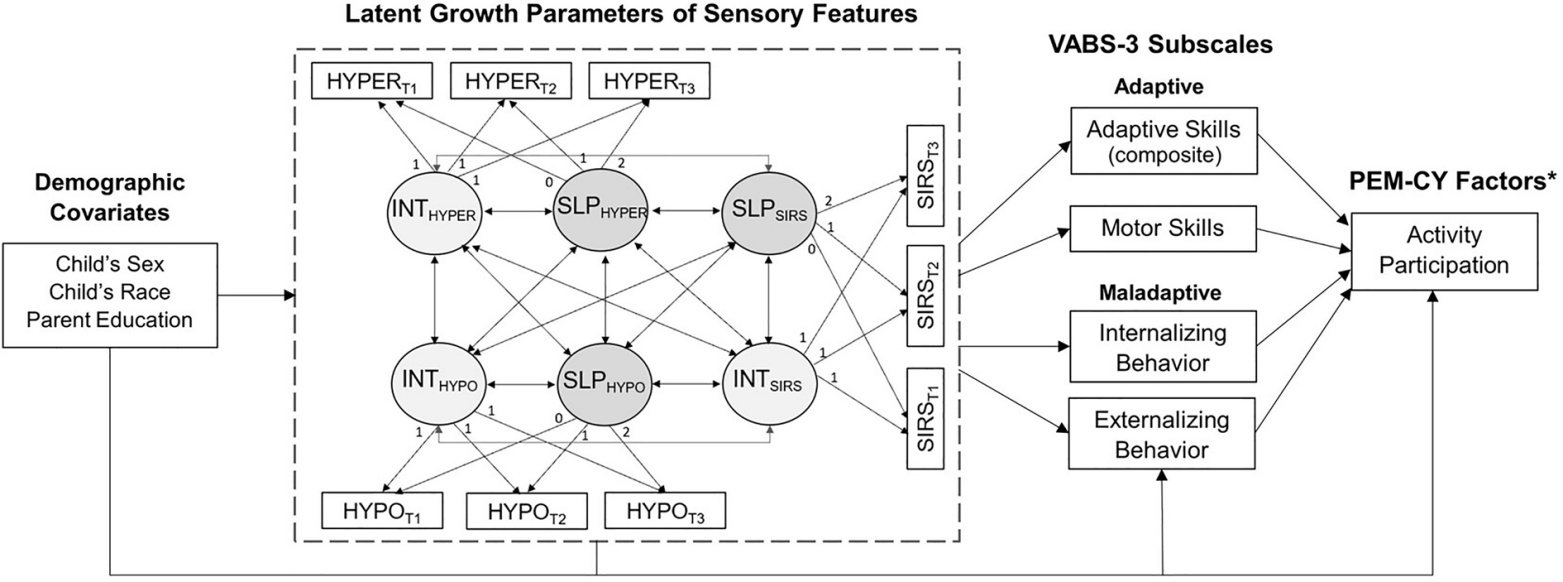
Hypothesized full structural equation model. The full model included a measurement part (as shown in the dashed square), which is a multivariate linear latent growth curve model for sensory patterns with factor loadings on the latent growth parameters (i.e., latent intercept and slope) and covariances among them. Additionally, the structural parts consist of regression paths estimating the associations between latent growth parameters and other observed variables (demographic covariates, VABS-3 and PEM-CY variables). Note *INT*  intercept, *SLP*  slope, *HYPER* hyperresponsiveness, *HYPO*  hyporesponsiveness, *SIRS* sensory repetitions/seeking behavior. *The model was fitted for each of the three PEM-CY settings: home (3 factors: routines/duties, screen-based activities, non-screen-based activities), school (2 factors: classroom-based and extracurricular activities), and community (3 factors: daily outings, unstructured physical activities, structured group activities)

To examine the multivariate associations among the observed and latent variables, the current hypothesized model (see Fig. 1) was built upon a multivariate linear latent growth curve model for estimating trajectories of sensory patterns across the three domains (HYPER, HYPO, and SIRS), which has been demonstrated as a good-fitting model in a previous study (χ^2^(15) = 27.44, CFI = 0.995, TLI = 0.988, RMSEA = 0.023; Chen et al., 2022b, 2024). The adaptive/maladaptive outcome variables were regressed onto the latent growth parameters of sensory patterns to assess the direct effects of sensory trajectories. Given the previous evidence that children’s activity participation can be influenced by their functional levels and behavioral challenges (Askari et al., 2015; Taheri et al., 2017), the participation outcomes were further regressed onto the adaptive/maladaptive outcome variables by each setting separately. This allowed for the examination of the direct and indirect effects of sensory trajectories on participation outcomes, while considering children’s level of adaptive functioning and maladaptive behavior. Additionally, three demographic variables (i.e., child’s sex, race/ethnicity, and parental education level) were added as covariates to account for their potential effects on the above-mentioned associations. The intercorrelations of the school-age outcome variables are shown in Table S2.

Further, an iterative approach was employed to assess the relative importance of predictors and explanatory variables for school-age outcomes. As shown in Figure S2, each group of predicting variables was added iteratively, and the change in R-squared (ΔR^2^) was evaluated to determine the additional variances explained by each predictive variable group across autistic and non-autistic groups using multigroup analysis. This allowed us to examine whether certain groups of predictors, such as sensory trajectories, accounted for more unique variances in participation outcomes among children classified as autistic compared to non-autistic. The structural equation modeling analyses (including latent growth curve modeling) were conducted with full-information maximum-likelihood estimation for handling missingness in Mplus 8.4 (Muthén & Muthén, 2018).

## Results

**Fig. 2:**
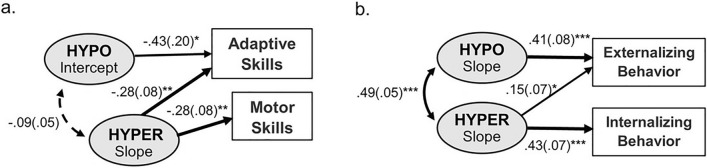
Differential effects of sensory trajectories on **a** adaptive and **b** maladaptive outcomes. Note. The displayed paths are subsets of the full model illustrated in Fig. 1. The values are standardized beta coefficients with corresponding standard errors. *p < .05, **p < .01, ***p < .001

The model fit statistics indicated good fits of the full models (CFI = 0.988 to 0.989, TLI = 0.958 to 0.960, RMSEA = 0.024 to 0.025; Table S3). The effects of sensory trajectories on adaptive/maladaptive outcomes controlling for demographic variables were presented in Table S4, with significant results visualized in Fig. 2. The greater slope (i.e., steeper increase) of HYPER predicted lower adaptive and motor skills (both β = -0.28, SE = 0.08, *p* = 0.001), as well as higher levels of internalizing behavior (β = 0.43, SE = 0.07, *p* < 0.001) and externalizing behavior (β = 0.15, SE = 0.07, *p* = 0.041) by school age. The higher HYPO at baseline predicted lower adaptive skills (β = -0.43, SE = 0.20, *p* = 0.034), and its steeper increase predicted more externalizing behavior (β = 0.41, SE = 0.08, *p* < 0.001). The latent growth parameters of SIRS were not significant predictors of any adaptive/maladaptive outcomes. The total variances of adaptive/maladaptive outcomes as explained by sensory trajectories and demographics ranged from 17.5% to 34.5%.

**Fig. 3:**
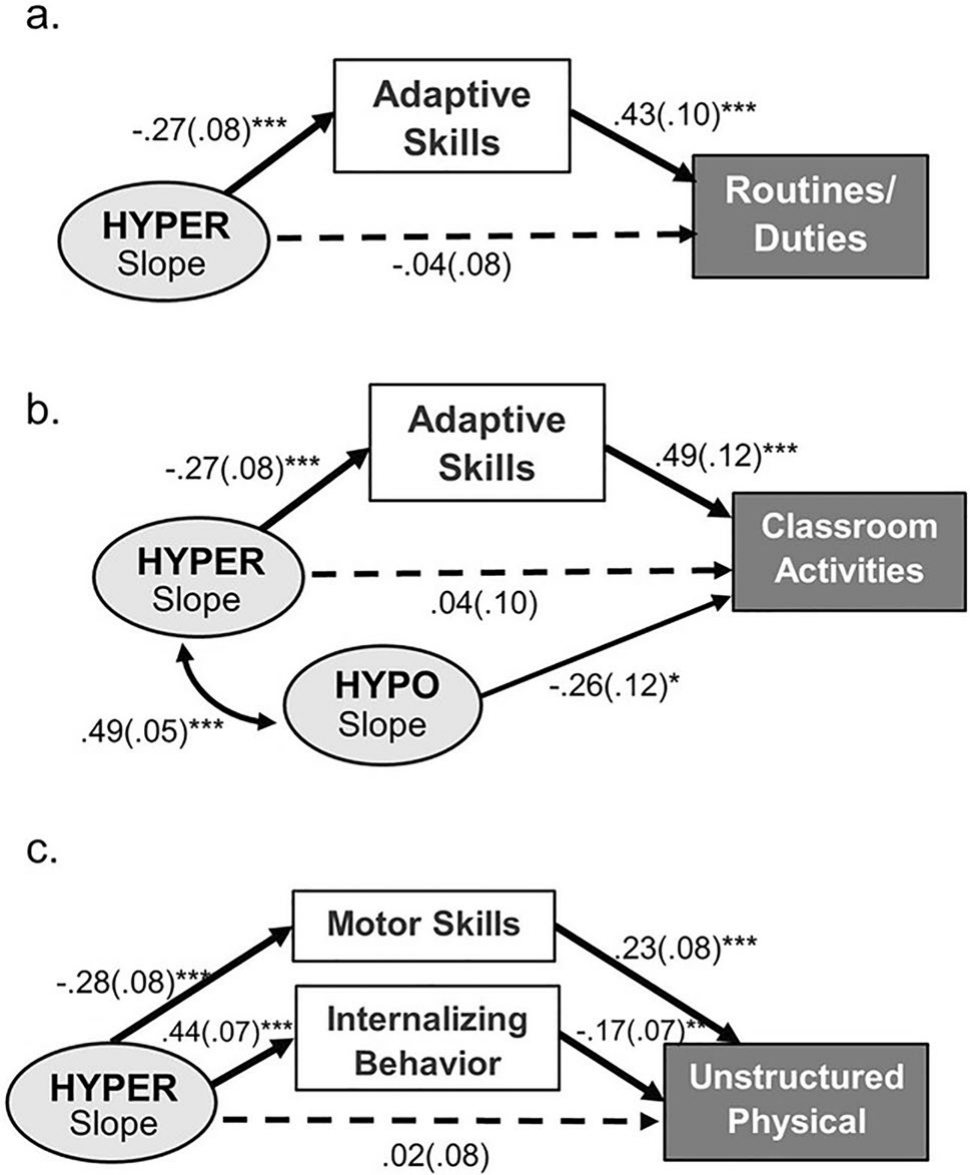
Differential effects of sensory trajectories on participation outcomes: **a** Indirect effect of sensory hyperresponsiveness on participation in routine/duty activities at home; **b** Indirect and direct effects of sensory hyperresponsiveness and hyporesponsiveness on participation in classroom activities at school; **c** Indirect effect of sensory hyperresponsiveness on participation in unstructured physical activities at community. Note. The displayed paths are subsets of the full model illustrated in Fig. 1. The values are standardized beta coefficients with corresponding standard errors. *p < .05, **p < .01, ***p < .001

The total effects of the predictors on participation outcomes across settings are reported in Table S5. Controlling for the effects of demographic variables, the steeper increase of HYPER indirectly predicted lower participation in certain categories of activities through the mediation of certain adaptive/maladaptive outcomes. As shown in Fig. 3, the effects of the slope of HYPER on participation in routines/duties and classroom-based activities were mediated by adaptive skills (β = -0.12 & -0.14, both SE = 0.05, *p* = 0.016 & 0.021), while its effects on unstructured physical activities were mediated by motor skills (β = -0.08, SE = 0.03, *p* = 0.018) and internalizing behavior (β = -0.06, SE = 0.03, *p* = 0.036). In other words, the steeper increase of HYPER was associated with lower adaptive and motor skills, which in turn respectively predicted lower participation outcomes in the aforementioned areas. Additionally, the steeper increase of HYPO was found to directly predict lower participation in classroom-based activities (β = -0.26, SE = 0.12, *p* = 0.033), without being mediated by adaptive or maladaptive behavior. The growth parameters of SIRS were not significant predictors of any participation outcomes. The total variances of participation outcomes as explained by all the predictors ranged from 6.5% to 36.1% across activity categories.

**Fig. 4:**
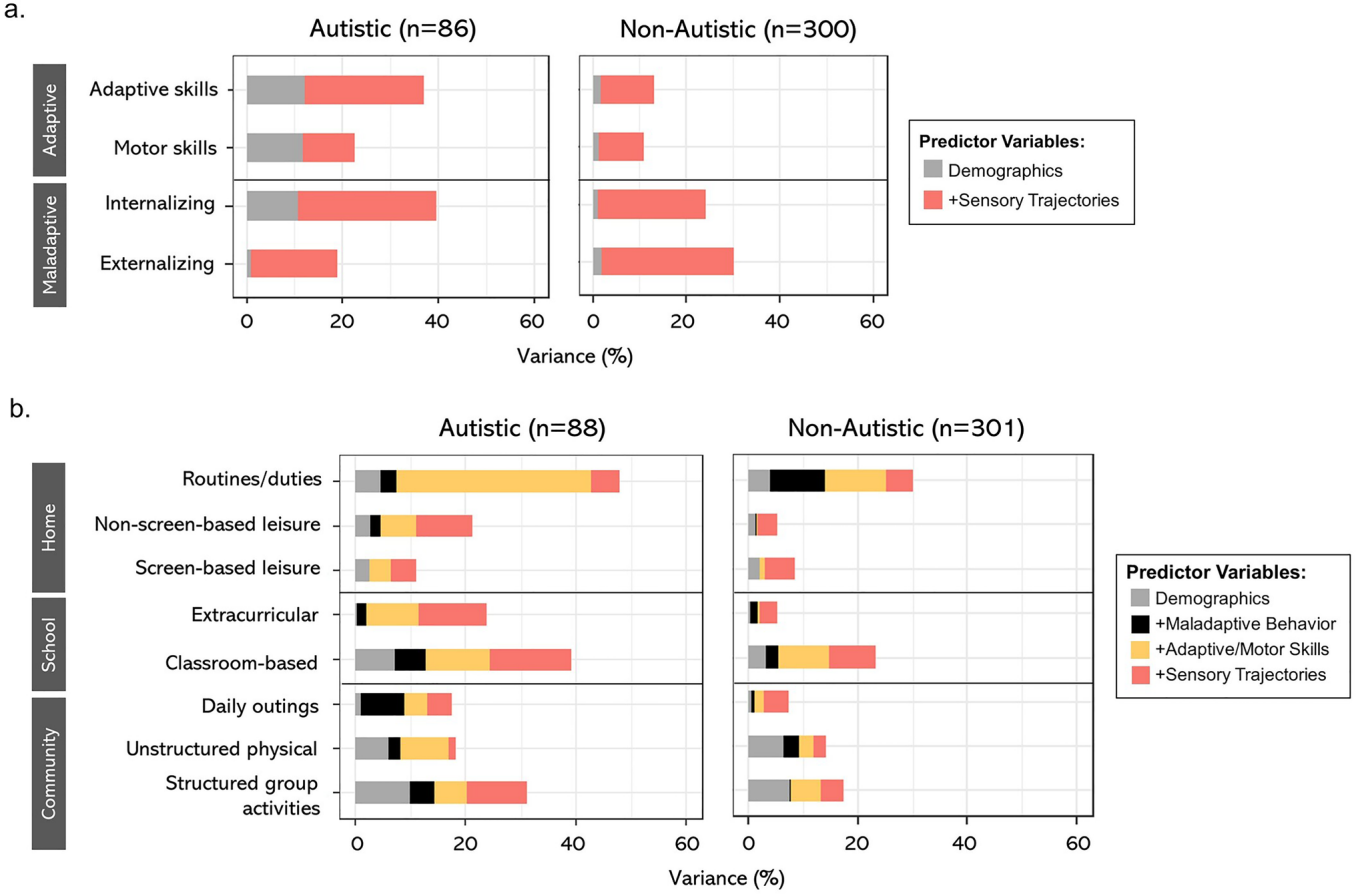
Explained variances (ΔR^2^) of adaptive/maladaptive and participation outcomes for the autistic and non-autistic groups: **a** VABS-3 adaptive/maladaptive outcomes; **b** PEM-CY participation outcomes

The multigroup models revealed that sensory trajectories explained 10.9–28.9% variances of adaptive/maladaptive outcomes beyond demographics for the autistic group, and 9.7–28.3% variances for the non-autistic group (see Fig. 4a). Specifically, sensory trajectories explained substantial portions of variances in adaptive skills (24.8%), internalizing behavior (28.9%), and externalizing behavior (18.2%) for the autistic group, and in externalizing (28.3%) and internalizing behavior (23.1%) for the non-autistic group. As for participation outcomes, all the predictors accounted for 11.1–47.9% variances of participation across activity categories for the autistic group, and 5.2–30.1% variances for the non-autistic group (see Fig. 4b). Specifically, sensory trajectories accounted for an additional 1.2–14.6% of the explained variances in participation outcomes above and beyond other predictors for the autistic group, and 2.2–8.5% for the non-autistic group. For children in the autistic group, sensory trajectories contributed to more than 10% of the explained variances in participation in classroom-based activities (14.6%), structured group activities in the community (10.8%), and non-screen-based leisure activities (10.3%).

## Discussion

This prospective study represents the first comprehensive, multivariate investigation into the developmental impacts of sensory patterns, including sensory hyperresponsiveness, hyporesponsiveness, and sensory repetitions/seeking behavior, on school-age adaptive, maladaptive, and participation outcomes in a birth cohort sample of autistic and non-autistic children. Our findings demonstrated that the early development of sensory patterns, particularly hyperresponsiveness and hyporesponsiveness, are differentially associated with specific outcome domains. The multigroup analysis further revealed the relative importance of early sensory development for predicting autistic children’s adaptive functioning and participation in daily life settings with higher functional and environmental demands. Below, we delve into a detailed discussion of the differential effects of sensory trajectories on the respective school-age outcome domains.

### Impacts of Sensory Trajectories on Adaptive Outcomes

Previous longitudinal studies, although few in number, have shown that early sensory differences may be negatively associated with later social communication (Grzadzinski et al., 2020; Worthley et al., 2023), daily living (Williams et al., 2018; Worthley et al., 2023), and motor skills (Worthley et al., 2023) for autistic children and infants at elevated likelihood for autism. However, these studies were limited in addressing how *change* in sensory patterns was associated with later outcomes. Through modeling within-person trajectories of sensory patterns, our current study reveals that elevated sensory *hypo*responsiveness at baseline (i.e., infancy), coupled with intensifying sensory *hyper*responsiveness during the preschool period, may cascade into later difficulties in social-adaptive and motor functioning. Notably, such cascading effects on school-age adaptive functioning appeared to be more pronounced among autistic children compared to non-autistic children. These findings support the cascading theory of sensory patterns on social development (Baranek et al., 2018; Thye et al., 2018) and highlight the importance of early detection and intervention in addressing sensory needs of young children with elevated hyperresponsive and hyporesponsive patterns in response to the sensory environment. By recognizing the intricate interplay of these sensory patterns and their changes over time, it may be possible to mitigate the potential negative impacts of sensory differences on children’s acquisition of adaptive skills through the provision of tailored interventions and supports for children’s sensory needs early in life (Chen et al., 2022a, 2022b; Watson et al., 2017).

### Impacts of Sensory Trajectories on Maladaptive Outcomes

The link between sensory hyperresponsiveness and internalizing behavior, such as anxiety, has been well supported by both behavioral and neurological evidence (Ben-Sasson et al., 2009; Carpenter et al., 2019; Green & Ben-Sasson, 2010; Green et al., 2012). Our findings align with the literature by demonstrating the specific contribution of sensory hyperresponsiveness to internalizing behavior, as a larger increase in hyperresponsiveness over time predicted higher levels of internalizing behavior at school age. Interestingly, while increasing *hyper*responsiveness was also associated with externalizing behavior, increasing *hypo*responsiveness emerged as a more significant predictor of externalizing behavior. The association between sensory patterns and externalizing behavior, as opposed to internalizing behavior, has received less attention in literature. Previous physiological evidence underscores a possible association between under-arousal and externalizing behavior in autistic children (Baker et al., 2018), suggesting a potential underlying mechanism warranting investigation in future research. The current finding adds behavioral evidence by demonstrating the differential roles of sensory hyperresponsiveness and hyporesponsiveness to later maladaptive behavior. Moreover, our multigroup analysis revealed that sensory trajectories accounted for a significant portion of variance in maladaptive outcomes for both autistic and non-autistic children. These findings suggest that early sensory differences could serve as a potential intervention target to mitigate the risk of cascading into maladaptive behavior and promote positive socio-emotional development across the neurodevelopmental spectrum. Moreover, it is important that clinical practitioners consider specific behavioral domains when evaluating developmental progress or the effectiveness of interventions (Chen et al., 2022a; Talbott & Miller, 2020).

### Impacts of Sensory Trajectories on Participation Outcomes Across Settings

Compared to adaptive/maladaptive outcome domains, sensory trajectories accounted for a smaller portion of variance in participation outcomes across settings, with some of their effects mediated by adaptive functioning or maladaptive behavior. This observation aligns with expectations, considering participation is viewed as a more distal functional outcome. As shown in Fig. 3b, a larger increase in *hypo*responsiveness was found directly associated with lower classroom participation. This longitudinal evidence adds to the prior cross-sectional finding of a concurrent association between hyporesponsiveness and academic performance (Ashburner et al., 2008). Further, a greater increase in *hyper*responsiveness was indirectly associated with lower participation in routine activities (e.g., personal care, doing homework) at home, classroom activities, and unstructured physical activities (e.g., playing sports, hanging out with peers) in the community. These results build on the previous longitudinal evidence that higher sensory hyperresponsiveness is associated with lower participation in routine activities at home and community activities among autistic children (Little et al., 2015). This suggests that the impact of sensory patterns on participation may be more pronounced in settings with greater functional and environmental demands, such as in the classroom and community. Thus, children with sensory differences may require additional support in these settings.

The observed mediating role of adaptive and motor skills between sensory development and participation outcomes may be explained by early-life sensory challenges imposing constraints on families, which lead to avoidance of certain stress-inducing learning opportunities, thereby restricting skill acquirement and adaptive growth. Over time, such constraints may further influence broader aspects of daily-life participation. Regarding the mediation of maladaptive behavior, we only observed internalizing behavior, along with motor skills, as a significant mediator between the change rate of *hyper*responsiveness and participation in unstructured physical activities. Anxiety related to sensorimotor differences can intensify in unstructured settings with less predictability, leading to avoidance and reduced participation in autistic and non-autistic populations (Ambrose et al., 2022; Arnell et al., 2020; Cairney et al., 2013). These findings thus highlight the importance of considering individual children’s sensory and developmental profiles when implementing environmental adaptations or sensory-based support strategies to facilitate children’s participation in various daily life settings (Pfeiffer et al., 2017).

Importantly, sensory trajectories overall accounted for a larger proportion of variance in participation outcomes for autistic children than for non-autistic children, particularly in non-screen-based leisure activities, classroom or extracurricular activities, and community group activities. This suggests that sensory differences may have a greater impact on the participation of autistic children in these specific activity areas compared to their non-autistic peers, thus underscoring the importance of addressing sensory needs through targeted interventions and support to promote inclusive participation for autistic children in these settings.

Finally, we found no significant association between sensory repetitions/seeking behavior and any of the school outcome domains. Previous literature has shown mixed evidence for associations between sensory repetitions/seeking behaviors and specific types of adaptive or participation outcomes (Hochhauser & Engel-Yeger, 2010; Kirby et al., 2019; Little et al., 2015; Watson et al., 2011; Williams et al., 2018). For instance, previous longitudinal evidence suggested negative associations between sensory repetitions/seeking behavior and adaptive functioning as well as community participation in autistic children, contrasting with positive associations observed in children with developmental delay (Kirby et al., 2019; Williams et al., 2018). It is possible that sensory repetitions/seeking behavior may serve as self-regulation strategies or motivators for children to engage in shared family activities and routines (Bagby et al., 2012; Hochhauser & Engel-Yeger, 2010), which could have positive effects on their functional outcomes and participation. Nevertheless, this sensory pattern may present challenges in certain contexts, such as distracting children from effectively following commands or completing required tasks in structured learning environments, thereby negatively impacting their functional performance (Jones et al., 2020). Additional factors that may be attributed to the mixed findings include children’s cognitive levels, parental perceptions or expectations, and parental stress related to sensory-seeking behavior and their functional impacts in various contexts (Kirby et al., 2015, 2019; Piccardi & Gliga, 2022; Williams et al., 2018). Further research that considers these child, parental, and environmental characteristics is warranted to comprehensively understand the positive and negative impacts of sensory repetitions/seeking behavior on child functioning.

### Limitations and Future Directions

Despite these novel longitudinal findings, some caveats need to be considered. This large cohort study necessarily relied on parent-reported measures of sensory patterns, developmental, and clinical outcomes. While we were restricted in our capacity to validate parent-reported diagnoses with gold-standard tests, prior population-based studies have supported the utility of parent-reported outcomes in clinical classification (Kogan et al., 2018; Warnell et al., 2015). Further, previous studies have demonstrated that the results may vary depending on whether the sensory patterns were assessed through parent-reported measures or clinician-observational measures (Grzadzinski et al., 2020; Williams et al., 2018). Therefore, the current findings must be interpreted in light of these limitations. Future research should also consider adopting a multi-informant multi-method approach including observational measures to obtain a more comprehensive understanding of the impacts of sensory patterns on other behavioral domains. Moreover, because participation is a complex construct shaped by a multitude of factors, future research should consider including other variables, such as school/community characteristics, environmental support or barriers, children’s preferences, peer interactions, and caregivers’ expectations, to understand their interplay with sensory patterns and participation outcomes, and how tailored supports for sensory needs may mitigate the cascade of sensory patterns on children’s functional development.

## Supplementary Information

Below is the link to the electronic supplementary material.

Supplementary file1 (DOCX 661 kb)

## Data Availability

The research data from the NCCDS-2 study are not publicly accessible as the participants did not provide consent for its release to a national repository. Inquiries regarding de-identified data supporting the findings of the current study may be directed to the principal investigator (Dr. Grace T. Baranek) routed through the corresponding author.

## References

[CR1] Ahn, R. R., Miller, L. J., Milberger, S., & McIntosh, D. N. (2004). Prevalence of parents’ perceptions of sensory processing disorders among kindergarten children. *American Journal of Occupational Therapy,**58*(3), 287–293. 10.5014/ajot.58.3.287 10.5014/ajot.58.3.287 15202626

[CR2] Ambrose, K., Simpson, K., & Adams, D. (2022). The impact of anxiety on the participation of children on the autism spectrum. *Journal of Autism and Developmental Disorders,**52*(7), 2958–2969. 10.1007/s10803-021-05162-x 34196892 10.1007/s10803-021-05162-x

[CR3] American Psychiatric Association. (2013). *Diagnostic and statistical manual of mental disorders: DSM-5 (Vol. 5, No. 5)*. Washington, DC: American psychiatric association. 10.1176/appi.books.9780890425596

[CR4] Arnell, S., Jerlinder, K., & Lundqvist, L. O. (2020). Parents’ perceptions and concerns about physical activity participation among adolescents with autism spectrum disorder. *Autism,**24*(8), 2243–2255. 10.1177/1362361320942092 32713182 10.1177/1362361320942092 PMC7543004

[CR5] Ashburner, J., Ziviani, J., & Rodger, S. (2008). Sensory processing and classroom emotional, behavioral, and educational outcomes in children with autism spectrum disorder. *The American Journal of Occupational Therapy,**62*(5), 564–573. 10.5014/ajot.62.5.564 18826017 10.5014/ajot.62.5.564

[CR6] Askari, S., Anaby, D., Bergthorson, M., Majnemer, A., Elsabbagh, M., & Zwaigenbaum, L. (2015). Participation of children and youth with autism spectrum disorder: A scoping review. *Review Journal of Autism and Developmental Disorders,**2*, 103–114. 10.1007/s40489-014-0040-7

[CR7] Bagby, M. S., Dickie, V. A., & Baranek, G. T. (2012). How sensory experiences of children with and without autism affect family occupations. *The American Journal of Occupational Therapy,**66*(1), 78–86. 10.5014/ajot.2012.000604 22389942 10.5014/ajot.2012.000604 PMC3324972

[CR8] Baker, A. E., Lane, A., Angley, M. T., & Young, R. L. (2008). The relationship between sensory processing patterns and behavioural responsiveness in autistic disorder: A pilot study. *Journal of Autism and Developmental Disorders,**38*(5), 867–875. 10.1007/s10803-007-0459-0 17899349 10.1007/s10803-007-0459-0

[CR9] Baker, J. K., Fenning, R. M., Erath, S. A., Baucom, B. R., Moffitt, J., & Howland, M. A. (2018). Sympathetic under-arousal and externalizing behavior problems in children with autism spectrum disorder. *Journal of Abnormal Child Psychology,**46*(4), 895–906. 10.1007/s10802-017-0332-3 28736798 10.1007/s10802-017-0332-3 PMC5783799

[CR10] Baranek, G. T. (1999). *Sensory Experiences Questionnaire (SEQ)*. Unpublished manuscript, University of North Carolina at Chapel Hill.

[CR11] Baranek, G. T., Watson, L. R., Crais, E. R., Turner-Brown, L. & Reznick, J. S. (2013). *First Years Inventory (FYI) 3.1*. Chapel Hill, NC: University of North Carolina at Chapel Hill.

[CR12] Baranek, G. T., David, F. J., Poe, M. D., Stone, W. L., & Watson, L. R. (2006). Sensory Experiences Questionnaire: Discriminating sensory features in young children with autism, developmental delays, and typical development. *Journal of Child Psychology and Psychiatry,**47*(6), 591–601. 10.1111/j.1469-7610.2005.01546.x 16712636 10.1111/j.1469-7610.2005.01546.x

[CR13] Baranek, G. T., Sideris, J., Chen, Y. J., Crais, E. R., Turner-Brown, L., & Watson, L. R. (2022). Early measurement of autism risk constructs in the general population: A new factor structure of the First Years Inventory (FYIv3.1) for ages 6–16 months. *Autism Research,**15*(5), 915–928. 10.1002/aur.2691 35243807 10.1002/aur.2691 PMC9314682

[CR14] Baranek, G. T., Woynaroski, T. G., Nowell, S., Turner-Brown, L., DuBay, M., Crais, E. R., & Watson, L. R. (2018). Cascading effects of attention disengagement and sensory seeking on social symptoms in a community sample of infants at-risk for a future diagnosis of autism spectrum disorder. *Developmental Cognitive Neuroscience,**29*, 30–40. 10.1016/j.dcn.2017.08.006 28869201 10.1016/j.dcn.2017.08.006 PMC6414208

[CR15] Ben-Sasson, A., Carter, A. S., & Briggs-Gowan, M. J. (2009). Sensory over-responsivity in elementary school: Prevalence and social-emotional correlates. *Journal of Abnormal Child Psychology,**37*(5), 705–716. 10.1007/s10802-008-9295-8 19153827 10.1007/s10802-008-9295-8 PMC5972374

[CR16] Ben-Sasson, A., Gal, E., Fluss, R., Katz-Zetler, N., & Cermak, S. A. (2019). Update of a meta-analysis of sensory symptoms in ASD: A new decade of research. *Journal of Autism and Developmental Disorders,**49*(12), 4974–4996. 10.1007/s10803-019-04180-0 31501953 10.1007/s10803-019-04180-0

[CR17] Boyd, B. A., Baranek, G. T., Sideris, J., Poe, M. D., Watson, L. R., Patten, E., & Miller, H. (2010). Sensory features and repetitive behaviors in children with autism and developmental delays. *Autism Research,**3*, 78–87. 10.1002/aur.124 20437603 10.1002/aur.124 PMC3071028

[CR18] Cairney, J., Rigoli, D., & Piek, J. (2013). Developmental coordination disorder and internalizing problems in children: The environmental stress hypothesis elaborated. *Developmental Review,**33*(3), 224–238. 10.1016/j.dr.2013.07.002

[CR19] Carpenter, K. L., Baranek, G. T., Copeland, W. E., Compton, S., Zucker, N., Dawson, G., & Egger, H. L. (2019). Sensory over-responsivity: An early risk factor for anxiety and behavioral challenges in young children. *Journal of Abnormal Child Psychology,**47*, 1075–1088. 10.1007/s10802-018-0502-y 30569253 10.1007/s10802-018-0502-y PMC6508996

[CR20] Chen, Y. J., Duku, E., & Georgiades, S. (2022a). Rethinking autism intervention science: A dynamic perspective. *Frontiers in Psychiatry,**13*, 827406. 10.3389/fpsyt.2022.827406 35280173 10.3389/fpsyt.2022.827406 PMC8915252

[CR21] Chen, Y. J., Sideris, J., Watson, L. R., Crais, E. R., & Baranek, G. T. (2022b). Developmental trajectories of sensory patterns from infancy to school age in a community sample and associations with autistic traits. *Child Development,**93*(4), e446–e459. 10.1111/cdev.13745 35238019 10.1111/cdev.13745

[CR22] Chen, Y. J., Sideris, J., Watson, L. R., Crais, E. R., & Baranek, G. T. (2024). Early developmental profiles of sensory features and links to school-age adaptive and maladaptive outcomes: A birth cohort investigation. *Development and Psychopathology,**36*(1), 291–301. 36579629 10.1017/S0954579422001195 PMC10307924

[CR23] Constantino, J. N., & Gruber, C. P. (2012). *Social Responsiveness Scale-Second Edition (SRS-2)*. Western Psychological Services.

[CR24] Coster, W., Bedell, G., Law, M., Khetani, M. A., Teplicky, R., Liljenquist, K., & Kao, Y. C. (2011). Psychometric evaluation of the participation and environment measure for children and youth. *Developmental Medicine & Child Neurology,**53*(11), 1030–1037. 10.1111/j.1469-8749.2011.04094.x 22014322 10.1111/j.1469-8749.2011.04094.x

[CR25] Coster, W., Law, M., & Bedell, G. (2010). *Participation and environment measure for children and youth*. Boston University.

[CR26] Dellapiazza, F., Michelon, C., Oreve, M. J., Robel, L., Schoenberger, M., Chatel, C., & Baghdadli, A. (2020). The impact of atypical sensory processing on adaptive functioning and maladaptive behaviors in autism spectrum disorder during childhood: Results from the ELENA cohort. *Journal of Autism and Developmental Disorders,**50*(6), 2142–2152. 10.1007/s10803-019-03970-w 30868365 10.1007/s10803-019-03970-w

[CR27] Feldman, J. I., Cassidy, M., Liu, Y., Kirby, A. V., Wallace, M. T., & Woynaroski, T. G. (2020). Relations between sensory responsiveness and features of autism in children. *Brain Sciences,**10*(11), 775. 10.1007/s10803-010-1007-x 33114357 10.3390/brainsci10110775 PMC7690864

[CR28] Green, S. A., & Ben-Sasson, A. (2010). Anxiety disorders and sensory over-responsivity in children with autism spectrum disorders: Is there a causal relationship? *Journal of Autism and Developmental Disorders,**40*(12), 1495–1504. 10.1007/s10803-010-1007-x 20383658 10.1007/s10803-010-1007-x PMC2980623

[CR29] Green, S. A., Ben-Sasson, A., Soto, T. W., & Carter, A. S. (2012). Anxiety and sensory over-responsivity in toddlers with autism spectrum disorders: Bidirectional effects across time. *Journal of Autism and Developmental Disorders,**42*(6), 1112–1119. 10.1007/s10803-011-1361-3 21935727 10.1007/s10803-011-1361-3 PMC4199633

[CR30] Grzadzinski, R., Donovan, K., Truong, K., Nowell, S., Lee, H., Sideris, J., & Watson, L. R. (2020). Sensory reactivity at 1 and 2 years old is associated with ASD severity during the preschool years. *Journal of Autism and Developmental Disorders,**50*(11), 3895–3904. 10.1007/s10803-020-04432-4 32157566 10.1007/s10803-020-04432-4 PMC7483928

[CR31] Hershberger, S. L. (2005). Factor scores. In B. S. Everitt & D. C. Howell (Eds.), *Encyclopedia of statistics in behavioral science* (pp. 636–644). Wiley.

[CR32] Hochhauser, M., & Engel-Yeger, B. (2010). Sensory processing abilities and their relation to participation in leisure activities among children with high-functioning autism spectrum disorder (HFASD). *Research in Autism Spectrum Disorders,**4*(4), 746–754. 10.1016/j.rasd.2010.01.015

[CR33] Jasmin, E., Couture, M., McKinley, P., Reid, G., Fombonne, E., & Gisel, E. (2009). Sensori-motor and daily living skills of preschool children with autism spectrum disorders. *Journal of Autism and Developmental Disorders,**39*(2), 231–241. 10.1007/s10803-008-0617-z 18629623 10.1007/s10803-008-0617-z

[CR34] Jones, E. K., Hanley, M., & Riby, D. M. (2020). Distraction, distress and diversity: Exploring the impact of sensory processing differences on learning and school life for pupils with autism spectrum disorders. *Research in Autism Spectrum Disorders,**72*, 101515. 10.1016/j.rasd.2020.101515

[CR35] Jussila, K., Junttila, M., Kielinen, M., Ebeling, H., Joskitt, L., Moilanen, I., & Mattila, M. L. (2020). Sensory abnormality and quantitative autism traits in children with and without autism spectrum disorder in an epidemiological population. *Journal of Autism and Developmental Disorders,**50*, 180–188. 10.1007/s10803-019-04237-0 31583623 10.1007/s10803-019-04237-0 PMC6946727

[CR36] Kirby, A. V., Bilder, D. A., Wiggins, L. D., Hughes, M. M., Davis, J., Hall-Lande, J. A., & Bakian, A. V. (2022). Sensory features in autism: Findings from a large population-based surveillance system. *Autism Research,**15*(4), 751–760. 10.1002/aur.2670 35040592 10.1002/aur.2670 PMC9067163

[CR37] Kirby, A. V., Little, L. M., Schultz, B., & Baranek, G. T. (2015). Observational characterization of sensory interests, repetitions, and seeking behaviors. *The American Journal of Occupational Therapy,**69*(3), 6903220010p1-6903220010p9. 10.5014/ajot.2015.015081 25871592 10.5014/ajot.2015.015081 PMC5362027

[CR38] Kirby, A. V., Williams, K. L., Watson, L. R., Sideris, J., Bulluck, J., & Baranek, G. T. (2019). Sensory features and family functioning in families of children with autism and developmental disabilities: Longitudinal associations. *The American Journal of Occupational Therapy,**73*(2), 7302205040p1-7302205040p14. 10.5014/ajot.2018.027391 30915965 10.5014/ajot.2018.027391 PMC6436113

[CR39] Kogan, M. D., Vladutiu, C. J., Schieve, L. A., Ghandour, R. M., Blumberg, S. J., Zablotsky, B., & Lu, M. C. (2018). The prevalence of parent-reported autism spectrum disorder among US children. *Pediatrics*. 10.1542/peds.2017-4161 30478241 10.1542/peds.2017-4161 PMC6317762

[CR40] Lee, H., Chen, Y. J., Sideris, J., Watson, L. R., Crais, E. R., & Baranek, G. T. (2022). Sensory features of young children from a large community sample: Latent factor structures of the Sensory Experiences Questionnaire (Version 2.1, Short Form). *American Journal of Occupational Therapy*. 10.5014/ajot.2022.046995 10.5014/ajot.2022.046995 35648120

[CR41] Liss, M., Saulnier, C., Fein, D., & Kinsbourne, M. (2006). Sensory and attention abnormalities in autistic spectrum disorders. *Autism,**10*, 155–172. 10.1177/1362361306062021 16613865 10.1177/1362361306062021

[CR42] Little, L. M., Ausderau, K., Sideris, J., & Baranek, G. T. (2015). Activity participation and sensory features among children with autism spectrum disorders. *Journal of Autism and Developmental Disorders,**45*(9), 2981–2990. 10.1007/s10803-015-2460-3 25975628 10.1007/s10803-015-2460-3 PMC6452625

[CR43] Little, L. M., Freuler, A. C., Houser, M. B., Guckian, L., Carbine, K., David, F. J., & Baranek, G. T. (2011). Psychometric validation of the sensory experiences questionnaire. *American Journal of Occupational Therapy,**65*, 207–210. 10.5014/ajot.2011.000844 10.5014/ajot.2011.000844 PMC3163482 21476368

[CR44] McCormick, C., Hepburn, S., Young, G. S., & Rogers, S. J. (2016). Sensory symptoms in children with autism spectrum disorder, other developmental disorders and typical development: A longitudinal study. *Autism,**20*(5), 572–579. 10.1177/1362361315599755 26395236 10.1177/1362361315599755 PMC4918912

[CR45] Meera, S. S., Donovan, K., Wolff, J. J., Zwaigenbaum, L., Elison, J. T., Kinh, T., & Network, I. B. I. S. (2020). Towards a data-driven approach to screen for autism risk at 12 months of age. *Journal of the American Academy of Child & Adolescent Psychiatry,**60*, 968–977. 10.1016/j.jaac.2020.10.015 33161063 10.1016/j.jaac.2020.10.015 PMC8127075

[CR46] Muthén, L. K., & Muthén, B. (2018). *Mplus. The comprehensive modelling program for applied researchers: User’s guide* (5th ed.). Muthén & Muthén.

[CR47] O’Donnell, S., Deitz, J., Kartin, D., Nalty, T., & Dawson, G. (2012). Sensory processing, problem behavior, adaptive behavior, and cognition in preschool children with autism spectrum disorders. *American Journal of Occupational Therapy,**66*(5), 586–594. 10.5014/ajot.2012.004168 10.5014/ajot.2012.004168 22917125

[CR48] Pfeiffer, B., Coster, W., Snethen, G., Derstine, M., Piller, A., & Tucker, C. (2017). Caregivers’ perspectives on the sensory environment and participation in daily activities of children with autism spectrum disorder. *The American Journal of Occupational Therapy,**71*(4), 7104220020p1-7104220028p9. 10.5014/ajot.2017.021360 28661385 10.5014/ajot.2017.021360 PMC5490458

[CR49] Piccardi, E. S., & Gliga, T. (2022). Understanding sensory regulation in typical and atypical development: The case of sensory seeking. *Developmental Review,**65*, 101037. 10.1016/j.dr.2022.101037

[CR50] Reynolds, S., Bendixen, R. M., Lawrence, T., & Lane, S. J. (2011). A pilot study examining activity participation, sensory responsiveness, and competence in children with high functioning autism spectrum disorder. *Journal of Autism and Developmental Disorders,**41*(11), 1496–1506. 10.1007/s10803-010-1173-x 21221753 10.1007/s10803-010-1173-x PMC5338357

[CR51] Reznick, J. S., Baranek, G. T., Watson, L. R., & Crais, E. R. (2005). *Developmental Concerns Questionnaire*. University of North Carolina.

[CR52] Riboldi, E. M., Capelli, E., Cantiani, C., Beretta, C., Molteni, M., & Riva, V. (2023). Differentiating early sensory profiles in toddlers at elevated likelihood of autism and association with later clinical outcome and diagnosis. *Autism*, 13623613231200081. 10.1177/13623613231200081 PMC11191663 37795823

[CR53] Sparrow, S. S., Cicchetti, D. V., & Balla, D. A. (2016). *Vineland-III: Vineland Adaptive Behavior Scales* (3rd ed.). Pearson.

[CR54] Surgent, O. J., Walczak, M., Zarzycki, O., Ausderau, K., & Travers, B. G. (2021). IQ and sensory symptom severity best predict motor ability in children with and without autism spectrum disorder. *Journal of Autism and Developmental Disorders,**51*, 243–254. 10.1007/s10803-020-04536-x 32410096 10.1007/s10803-020-04536-x PMC7665981

[CR55] Taheri, A., Perry, A., & Minnes, P. (2017). Exploring factors that impact activity participation of children and adolescents with severe developmental disabilities. *Journal of Intellectual Disability Research,**61*(12), 1151–1161. 10.1111/jir.12437 29154492 10.1111/jir.12437

[CR56] Talbott, M. R., & Miller, M. R. (2020). Future directions for infant identification and intervention for autism spectrum disorder from a transdiagnostic perspective. *Journal of Clinical Child & Adolescent Psychology,**49*(5), 688–700. 10.1080/15374416.2020.1790382 32701034 10.1080/15374416.2020.1790382 PMC7541743

[CR57] Thye, M. D., Bednarz, H. M., Herringshaw, A. J., Sartin, E. B., & Kana, R. K. (2018). The impact of atypical sensory processing on social impairments in autism spectrum disorder. *Developmental Cognitive Neuroscience,**29*, 151–167. 10.1016/j.dcn.2017.04.010 28545994 10.1016/j.dcn.2017.04.010 PMC6987885

[CR58] Tomchek, S. D., Little, L. M., & Dunn, W. (2015). Sensory pattern contributions to developmental performance in children with autism spectrum disorder. *American Journal of Occupational Therapy,**69*(5), 6905185040p1-6905185040p10. 10.5014/ajot.2015.018044 10.5014/ajot.2015.018044 26356661

[CR59] Turner-Brown, L. M., Baranek, G. T., Reznick, J. S., Watson, L. R., & Crais, E. R. (2013). The First Year Inventory: A longitudinal follow-up of 12-month-old to 3-year-old children. *Autism,**17*, 527–540. 10.1177/1362361312439633 22781058 10.1177/1362361312439633 PMC3470769

[CR60] Uljarević, M., Baranek, G., Vivanti, G., Hedley, D., Hudry, K., & Lane, A. (2017). Heterogeneity of sensory features in autism spectrum disorder: Challenges and perspectives for future research. *Autism Research,**10*(5), 703–710. 10.1002/aur.1747 28266796 10.1002/aur.1747

[CR61] Warnell, F., George, B., McConachie, H., Johnson, M., Hardy, R., & Parr, J. R. (2015). Designing and recruiting to UK autism spectrum disorder research databases: Do they include representative children with valid ASD diagnoses? *British Medical Journal Open,**5*(9), e008625. 10.1136/bmjopen-2015-008625 10.1136/bmjopen-2015-008625 PMC4577974 26341584

[CR62] Watson, L. R., Crais, E. R., Baranek, G. T., Turner-Brown, L., Sideris, J., Wakeford, L., & Nowell, S. W. (2017). Parent-mediated intervention for one-year-olds screened as at-risk for autism spectrum disorder: A randomized controlled trial. *Journal of Autism and Developmental Disorders,**47*, 3520–3540. 10.1007/s10803-017-3268-0 28861651 10.1007/s10803-017-3268-0

[CR63] Watson, L. R., Patten, E., Baranek, G. T., Poe, M., Boyd, B. A., Freuler, A., & Lorenzi, J. (2011). Differential associations between sensory response patterns and language, social, and communication measures in children with autism or other developmental disabilities. *Journal of Speech, Language, and Hearing Research,**54*(6), 1562–1576. 10.1044/1092-4388(2011/10-0029) 21862675 10.1044/1092-4388(2011/10-0029) PMC3325756

[CR64] Williams, K. L., Kirby, A. V., Watson, L. R., Sideris, J., Bulluck, J., & Baranek, G. T. (2018). Sensory features as predictors of adaptive behaviors: A comparative longitudinal study of children with autism spectrum disorder and other developmental disabilities. *Research in Developmental Disabilities,**81*, 103–112. 10.1016/j.ridd.2018.07.002 30060977 10.1016/j.ridd.2018.07.002 PMC7473611

[CR65] Wolff, J. J., Dimian, A. F., Botteron, K. N., Dager, S. R., Elison, J. T., Estes, A. M., & Network, I. B. I. S. (2019). A longitudinal study of parent-reported sensory responsiveness in toddlers at-risk for autism. *Journal of Child Psychology and Psychiatry,**60*, 314–324. 10.1111/jcpp.12978 30350375 10.1111/jcpp.12978 PMC8919956

[CR66] Worthley, E., Grzadzinski, R., Zwaigenbaum, L., Dager, S. R., Estes, A. M., Hazlett, H. C., & Network, I. B. I. S. (2023). Sensory profiles in relation to later adaptive functioning among toddlers at high-familial likelihood for autism. *Journal of Autism and Developmental Disorders*. 10.1007/s10803-022-05869-5 37017863 10.1007/s10803-022-05869-5 PMC11654832

