# Supplementary material for: Developmental Impacts of Early Sensory Patterns on School-Age Adaptive, Maladaptive, and Participation Outcomes in Autistic and Non-autistic Children
Source: J Autism Dev Disord. 2024 Aug 2;55(11):4033–44. doi: 10.1007/s10803-024-06494-0 (PMC12575585; doi:10.1007/s10803-024-06494-0)
Supplement: Supplementary file 1 — Supplementary file1 (DOCX 661 kb) [file 10803_2024_6494_MOESM1_ESM.docx]

**Supporting Information**

**Appendix.** Establishing item-response-theory (IRT) based trait scores of sensory patterns

The 14 sensory items extracted from the FYIv3.1 (measured at T1) and SEQv2.1 (measured at T2 & T3) across the three sensory constructs are listed below:

| **HYPER (5 items)** | **HYPO (4 items)** | **SIRS (5 items)** |
| --- | --- | --- |
| - Refuse certain food textures^ab^ | - Ignore when name called^ab^ | - Look at objects in unusual ways^*ab^ |
| - Avoid looking at face^ab^ | - Tune-out sounds/noises^*ab^ | - Fascinated with textures^a^ |
| - Distressed when touched^ab^ | - Slow to notice new objects^a^ | - Fascinated with lights^b^ |
| - Distressed at loud sounds^*ab^ | - Slow to react to pain^b^ | - Flap arms/hands^a^ |
| - Distressed during grooming^*a^ |  | - Mouth non-food objects^b^ |

^*^Anchor items; ^ab^common items across FYI-A/B form; ^a^items specific to FYI-A form; ^b^items specific to FYI-B form (Note: All the 14 items are available in the SEQ); HYPER=sensory hyper-responsiveness; HYPO=sensory hypo-responsiveness; SIRS=sensory interests, repetitions and seeking behaviors

To ensure the comparability of construct scores across measures and time-points (i.e., to meet the assumption of measurement invariance) before conducting latent growth modeling, we first tested whether longitudinal invariance held at the configural level over time, followed by metric and scalar invariance tests for each of the three sensory constructs^1^ with full-information maximum likelihood (FIML) estimation. Differences in fit indices between models were evaluated to determine whether invariance held at different levels. A decrease in comparative fit index (CFI) or Tucker-Lewis Index (TLI) >.01, or an increase in root-mean-square error of approximation (RMSEA) >.01 indicates measurement non-invariance.^2^ The purpose of invariance testing in the current study was to ensure that at least configural invariance was met before constructing IRT trait scores that adjust for differential item functioning (DIF). Longitudinal invariance testing on each of the constructs demonstrated invariance at least at the configural level (see below for the model fits), indicating that the constructs to be measured by the selected items held constant across time-points.

|  | HYPER | | | HYPO | | | SIRS | | |
| --- | --- | --- | --- | --- | --- | --- | --- | --- | --- |
|  | CFI | TLI | RMSEA | CFI | TLI | RMSEA | CFI | TLI | RMSEA |
| Configural | .955 | .934 | .039 | 1.00 | 1.00 | <.001 | .982 | .973 | .021 |
| Metric | .951 | .935 | .038 | .981 | .971 | .016 | .978 | .971 | .022 |
| Scalar | .719 | .662 | .087 | .571 | .420 | .072 | .767 | .722 | .067 |

Next, DIF was evaluated to determine which non-DIF items could be used as anchor items for scale equating, using a test-characteristic-curve equating procedure.^3^ A relatively conservative criterion (McFadden’s pseudo-R^2^ change ≥0.02 between nested logistic regression DIF models) was used to detect meaningful DIF.^4^ It has been recommended to have at least one anchor item for every four non-common items to avoid construct drift.^5^ As a result, we identified one to two anchor (non-DIF) items for each sensory construct with McFadden’s pseudo-R^2^ change ranging from .003 to .013. By recalibrating group-specific item parameter estimates (i.e., estimates specific to each time-point) for the DIF items, trait scores of HYPER, HYPO, and SIRS that accounted for DIF across time-points were generated. The DIF detection and trait score derivation were implemented with R package lordif.^6^ The descriptive statistics of the trait scores for the full sample are as below:

|  | N | Mean | SD | Min | Max | Skewness | Kurtosis |
| --- | --- | --- | --- | --- | --- | --- | --- |
| HYPER *T1* | 1,515 | .10 | .66 | -1.39 | 2.06 | .23 | 2.54 |
| *T2* | 1,507 | -.01 | .75 | -1.35 | 3.03 | .54 | 3.45 |
| *T3* | 1,508 | -.10 | .80 | -1.34 | 3.17 | .58 | 3.48 |
| HYPO *T1* | 1,517 | -.03 | .59 | -.90 | 1.93 | .41 | 2.48 |
| *T2* | 1,508 | .03 | .70 | -.94 | 3.01 | .37 | 2.96 |
| *T3* | 1,508 | .06 | .71 | -.86 | 2.79 | .42 | 2.85 |
| SIRS *T1* | 1,502 | .11 | .81 | -1.49 | 2.81 | .25 | 2.46 |
| *T2* | 1,507 | -.02 | .88 | -1.16 | 2.99 | .46 | 2.60 |
| *T3* | 1,508 | -.12 | .85 | -1.03 | 3.22 | .67 | 2.68 |

References:

1. Millsap, R. E. (2012). *Statistical Approaches to Measurement Invariance*. Routledge.
2. Cheung, G. W., & Rensvold, R. B. (2002). Evaluating goodness-of-fit indexes for testing measurement invariance. *Structural Equation Modeling*, *9*, 233-255. doi: 10.1207/s15328007sem0902_5.
3. Stocking, M. L., & Lord, F. M. (1983). Developing a common metric in item response theory. *Applied Psychological Measurement*, *7*, 201-210. doi: 10.1177/014662168300700208
4. Paz, S. H., Spritzer, K. L., Morales, L. S., & Hays, R. D. (2013). Evaluation of the patient-reported outcomes information system (PROMIS®) Spanish-language physical functioning items. *Quality of Life Research*, *22*, 1819-1830. doi: 10.1007/s11136-012-0292-6.
5. Kolen, M. J., Brennan, R. L., & Kolen, M. J. (2004). *Test Equating, Scaling, and Linking: Methods and Practices* (pp. 177-180). New York: Springer.
6. Choi, S. W., Gibbons, L. E., & Crane, P. K. (2016). Lordif: Logistic ordinal regression differential item functioning using IRT. https://CRAN.R-project.org/package=lordif, R package version 0.3-3.

Figure S1. Results of common factor analysis for the PEM-CY measure

a. Scree plots with parallel analysis (200 iterations)

**Home (10 items) School (5 items) Community (8 items)**


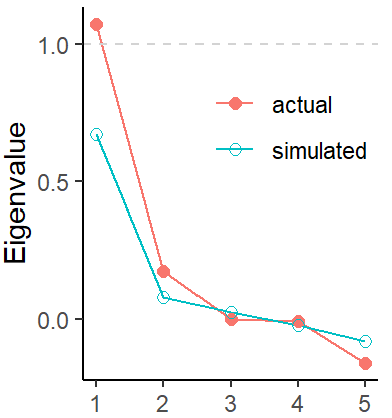


Number of Factors


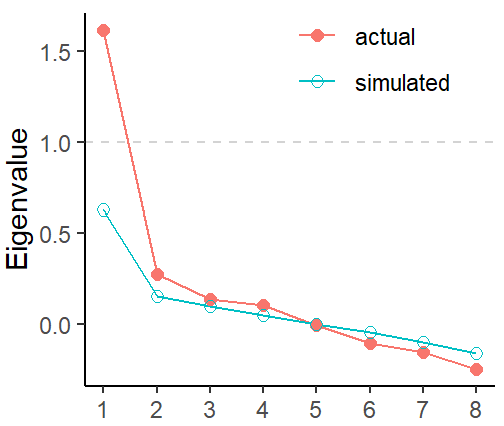

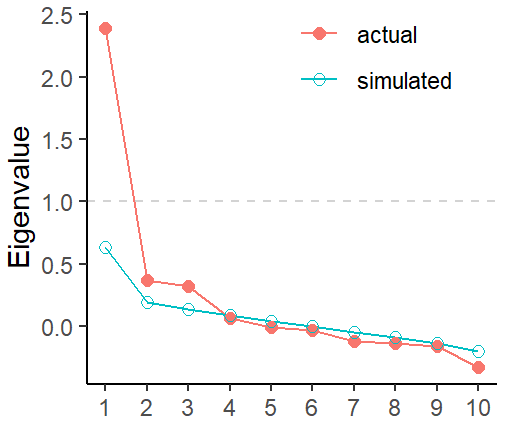


| Home | | | |
| --- | --- | --- | --- |
| Factor # | 2 | **3** | 4 |
| BIC | -91.42 | **-81.20** | -52.14 |
| TLI | .894 | **.967** | .984 |
| RMSEA | .061 | **.034** | .024 |

| School | | |
| --- | --- | --- |
| Factor # | 1 | **2** |
| BIC | -12.76 | **-4.59** |
| TLI | .826 | **.973** |
| RMSEA | .079 | **.031** |

| Community | | | |
| --- | --- | --- | --- |
| Factor # | 2 | **3** | 4 |
| BIC | -38.00 | **-28.05** | -7.73 |
| TLI | .814 | **.913** | .899 |
| RMSEA | .072 | **.050** | .053 |

Model fit statistics (with varimax rotation)

Note. Selected factor solution was bolded.

b. Factoring patterns

**Home (10 items) School (5 items) Community (8 items)**


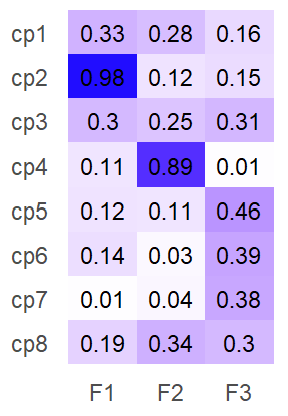


Neighborhood outing

Organized physical

Unstructured physical

Group/volunteer

Together w/ other kids

Community events

Lessons

Religious


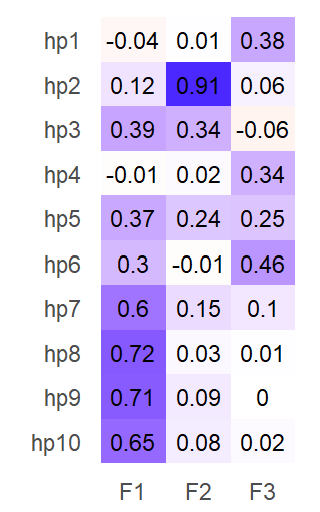


Computer games

Arts/hobbies

Social w/ tech

Personal care

Homework

Indoor play

Together w/ people

House chores

School preparation

Watching TV


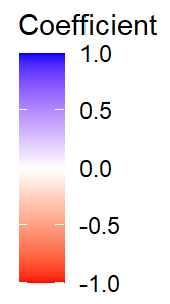


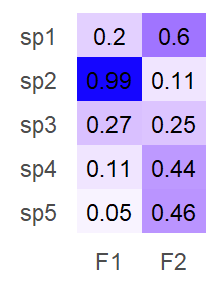


Classroom

Field trip/events

School clubs

Together w/ peers

Special roles

Note. Two items were dropped - "*Working for Pay*" and "*Overnight Trips*" due to rare endorsement.

|  | **Home** | **School** | **Community** |
| --- | --- | --- | --- |
| Factor 1 (F1) | Routines/duties (HP1) | Extracurricular activities (SP1) | Daily outings (CP1) |
| Factor 2  (F2) | Non-screen-based leisure activities (HP2) | Classroom-based activities (SP2) | Unstructured physical activities (CP2) |
| Factor 3 (F3) | Screen-based leisure activities (HP3) | -- | Structured group activities (CP3) |

c. Factor naming

Table S1. Descriptive statistics of PEM-CY factor scores by clinical outcome group

| PEM-CY Factors (Activity Types) | Autistic (N=88) | | | | Non-autistic (N=301) | | | |
| --- | --- | --- | --- | --- | --- | --- | --- | --- |
|  | Mean | SD | Skew. | Kurt. | Mean | SD | Skew. | Kurt. |
| HP1: Routines/duties | -.73 | 1.32 | -.17 | .00 | .21 | .98 | -.60 | .28 |
| HP2: Non-screen-based leisure | -.33 | 1.40 | -.91 | -.34 | .10 | .98 | -2.13 | 6.48 |
| HP3: Screen-based leisure | -.19 | 1.84 | .26 | .30 | .05 | 1.50 | -.15 | -.26 |
| SP1: Extracurricular | -.28 | 1.07 | 1.71 | 3.89 | .08 | .98 | .92 | 1.14 |
| SP2: Classroom-based | -.82 | 1.65 | -.41 | -.45 | .24 | 1.19 | -.76 | 1.38 |
| CP1: Daily outings | -.17 | .93 | .95 | .96 | .05 | 1.04 | .52 | -.23 |
| CP2: Unstructured physical | -.38 | 1.29 | -.62 | -.64 | .11 | 1.04 | -1.07 | .82 |
| CP3: Structured group | -.45 | 1.51 | .81 | -.08 | .13 | 1.50 | .59 | .19 |

Table S2. Intercorrelations of school-age outcome variables (N=389)

|  | 1 | 2 | 3 | 4 | 5 | 6 | 7 | 8 | 9 | 10 | 11 | 12 |
| --- | --- | --- | --- | --- | --- | --- | --- | --- | --- | --- | --- | --- |
| VABS |  |  |  |  |  |  |  |  |  |  |  |  |
| 1. ABC | -- |  |  |  |  |  |  |  |  |  |  |  |
| 2. MOT | .64 | -- |  |  |  |  |  |  |  |  |  |  |
| 3. INT | -.34 | -.32 | -- |  |  |  |  |  |  |  |  |  |
| 4. EXT | -.44 | -.27 | .59 | -- |  |  |  |  |  |  |  |  |
| PEM-CY |  |  |  |  |  |  |  |  |  |  |  |  |
| 5. HP1 | .55 | .44 | -.30 | -.33 | -- |  |  |  |  |  |  |  |
| 6. HP2 | .10 | .06 | -.12 | -.05 | -.05 | -- |  |  |  |  |  |  |
| 7. HP3 | .09 | .01 | .00 | -.02 | -.08 | -.04 | -- |  |  |  |  |  |
| 8. SP1 | .17 | .17 | -.14 | -.11 | .13 | .21 | .09 | -- |  |  |  |  |
| 9. SP2 | .47 | .33 | -.25 | -.31 | .46 | .17 | .01 | -.06 | -- |  |  |  |
| 10. CP1 | .17 | .15 | -.03 | -.12 | .18 | .03 | .08 | .33 | .15 | -- |  |  |
| 11. CP2 | .14 | .27 | -.27 | -.13 | .18 | .17 | .03 | .12 | .17 | -.01 | -- |  |
| 12. CP3 | .32 | .24 | -.16 | -.13 | .20 | .08 | .05 | .15 | .17 | -.12 | -.06 | -- |

ABC=adaptive behavior composite, MOT=motor skills, INT=internalizing behavior, EXT=externalizing behavior, HP=home participation, SP=school participation, CP=community participation.


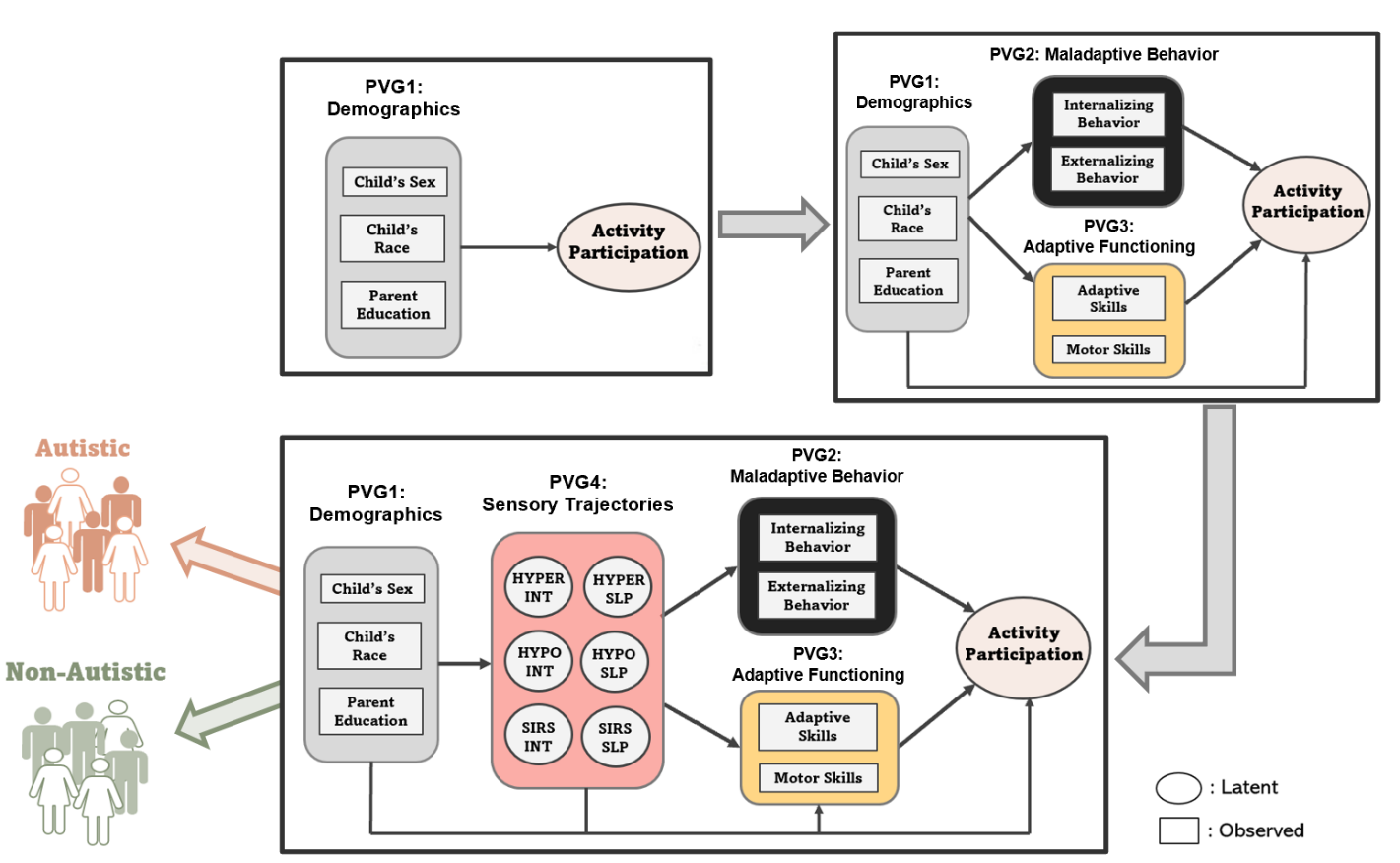
Figure S2. Iterative process for assessing the relative importance of explanatory variables on participation outcomes across autistic and non-autistic groups

The different predictor variable groups (PVG) were added iteratively to assess the change in explained variance (ΔR^2^) of participation outcomes in the sequence of demographics (PVG1), maladaptive behavior (PVG2), adaptive functioning (PVG3), and sensory trajectories (PVG4). For instance, by comparing the models with PVG1-3 versus with PVG1-4 included, we were able to assess the additional variance explained by sensory trajectories (PVG4) above and beyond other predictors. The whole process was implemented in multigroup models, where R^2^ values were available for each of the autistic and non-autistic groups. INT=intercept; SLP=slope

| Setting | χ^2^ | df | CFI | TLI | RMSEA [90% CI] |
| --- | --- | --- | --- | --- | --- |
| Home | 3758.04 | 168 | .989 | .960 | .024 [.016, .031] |
| School | 3785.35 | 150 | .988 | .960 | .025 [.017, .033] |
| Community | 3796.71 | 168 | .988 | .958 | .024 [.017, .032] |

Table S3. Model fit statistics of the hypothesized full model by setting

Table S4. Total effects of predicting variables on adaptive/maladaptive outcomes

|  | Adaptive Functioning | | | Motor Skills | | |
| --- | --- | --- | --- | --- | --- | --- |
|  | β | SE | *p* | β | SE | *p* |
| Sex | .206 | .047 | <.001^***^ | .144 | .048 | .003^**^ |
| Race | -.044 | .051 | .390 | .021 | .060 | .720 |
| Edu | .111 | .048 | .021^*^ | .166 | .049 | .001^**^ |
| Hyper *- Intercept* | .098 | .260 | .708 | -.289 | .226 | .201 |
| *Slope* | -.275 | .080 | .001^**^ | -.279 | .080 | .001^**^ |
| Hypo *- Intercept* | -.426 | .201 | .034^*^ | -.005 | .174 | .977 |
| *Slope* | -.085 | .095 | .368 | -.034 | .085 | .689 |
| SIRS *- Intercept* | .020 | .128 | .874 | .148 | .124 | .233 |
| *Slope* | .010 | .097 | .915 | -.065 | .088 | .460 |
| *Total variance explained (R^2^)* | 26.5% | | | 17.5% | | |

1. Adaptive outcomes

|  | **Internalizing Behavior** | | | **Externalizing Behavior** | | |
| --- | --- | --- | --- | --- | --- | --- |
|  | β | SE | *p* | β | SE | *p* |
| Sex | -.050 | .046 | .279 | -.161 | .045 | <.001^***^ |
| Race | .060 | .045 | .183 | .001 | .048 | .991 |
| Edu | -.151 | .044 | .001^**^ | -.060 | .045 | .182 |
| Hyper *- Intercept* | .342 | .184 | .063^†^ | .297 | .202 | .142 |
| *Slope* | .432 | .069 | <.001^***^ | .148 | .072 | .041^*^ |
| Hypo *- Intercept* | .059 | .148 | .691 | .164 | .171 | .338 |
| *Slope* | .072 | .073 | .323 | .414 | .081 | <.001^***^ |
| SIRS *- Intercept* | .000 | .107 | .997 | -.080 | .110 | .465 |
| *Slope* | -.016 | .073 | .828 | .052 | .073 | .478 |
| *Total variance explained (R^2^)* | 30.4% | | | 34.5% | | |

1. Maladaptive outcomes

Table S5. Total effects of predicting variables on participation outcomes across autistic and non-autistic samples (N=389) in the full model

|  | **Routines/Duties** | | | **Non-Screen-Based Leisure Activities** | | | **Screen-Based Leisure Activities** | | |
| --- | --- | --- | --- | --- | --- | --- | --- | --- | --- |
|  | β | SE | *p* | β | SE | *p* | Β | SE | *p* |
| Sex | .241 | .046 | <.001^***^ | .090 | .048 | .059^†^ | -.088 | .050 | .081 |
| Race | .055 | .051 | .287 | -.076 | .058 | .191 | .077 | .065 | .235 |
| Edu | .050 | .048 | .297 | .078 | .052 | .135 | .003 | .051 | .957 |
| Internalizing | -.082 | .059 | .165 | -.142 | .070 | .041^*^ | -.007 | .079 | .929 |
| Externalizing | .013 | .083 | .876 | .214 | .092 | .020^*^ | -.074 | .104 | .475 |
| Adaptive | .431 | .103 | <.001^***^ | .116 | .105 | .269 | .121 | .118 | .303 |
| Motor | .066 | .075 | .381 | -.056 | .090 | .531 | -.020 | .094 | .829 |
| Hyper *- Intercept* | -.255 | .222 | .250 | -.039 | .232 | .866 | .311 | .255 | .167 |
| *Slope* | -.209 | .080 | .009^**ind1^ | .075 | .080 | .346 | -.030 | .085 | .728 |
| Hypo *- Intercept* | -.092 | .167 | .581 | -.046 | .185 | .804 | -.177 | .189 | .350 |
| *Slope* | -.135 | .089 | .131 | -.195 | .090 | .031^*^ | .052 | .098 | .597 |
| SIRS *- Intercept* | .202 | .135 | .105 | -.038 | .124 | .758 | -.106 | .131 | .416 |
| *Slope* | .000 | .093 | .997 | -.016 | .085 | .852 | .015 | .086 | .862 |
| *Total variance explained (R^2^)* | 36.1% | | | 8.2% | | | 6.5% | | |

1. Home participation outcomes

ind1: indirect effect via adaptive skills (β=-.117, SE=.049, *p*=.016). ^*^*p* < .05, ^**^*p* < .01, ^***^*p* < .001.

|  | **Extracurricular Activities** | | | **Classroom-Based Activities** | | |
| --- | --- | --- | --- | --- | --- | --- |
|  | β | SE | *p* | β | SE | *p* |
| Sex | .055 | .052 | .292 | .142 | .047 | .003^**^ |
| Race | -.040 | .056 | .473 | -.065 | .059 | .269 |
| Edu | -.009 | .049 | .857 | .178 | .051 | <.001^***^ |
| Internalizing | -.096 | .076 | .209 | -.032 | .087 | .716 |
| Externalizing | .118 | .093 | .207 | .036 | .113 | .751 |
| Adaptive | .104 | .108 | .336 | .492 | .121 | <.001^***^ |
| Motor | .046 | .108 | .616 | -.079 | .091 | .389 |
| Hyper *- Intercept* | -.293 | .227 | .197 | -.338 | .233 | .146 |
| *Slope* | -.008 | .083 | .927 | -.081 | .083 | .334^ind2^ |
| Hypo *- Intercept* | -.002 | .192 | .992 | .052 | .184 | .779 |
| *Slope* | -.142 | .099 | .154 | -.282 | .091 | .002^**^ |
| SIRS *- Intercept* | .188 | .133 | .157 | .211 | .124 | .090 |
| *Slope* | -.054 | .090 | .548 | .011 | .094 | .909 |
| *Total variance explained (R^2^)* | 8.7% | | | 31.8% | | |

1. School participation outcomes

ind2: indirect effect via adaptive skills (β=-.135, SE=.059, *p*=.021). ^*^*p* < .05, ^**^*p* < .01, ^***^*p* < .001.

|  | **Daily Outings** | | | | **Unstructured Physical Activities** | | | **Structured Group Activities** | | |
| --- | --- | --- | --- | --- | --- | --- | --- | --- | --- | --- |
|  | β | | SE | *p* | β | SE | *p* | β | SE | *p* |
| Sex | .013 | .051 | | .803 | -.042 | .050 | .400 | .237 | .048 | <.001^***^ |
| Race | -.022 | .052 | | .669 | -.136 | .051 | .007^**^ | .033 | .049 | .497 |
| Edu | .030 | .051 | | .550 | .192 | .049 | <.001^***^ | .133 | .047 | .005^**^ |
| Internalizing | .129 | .077 | | .095 | -.173 | .066 | .009^**^ | -.040 | .070 | .563 |
| Externalizing | -.159 | .096 | | .097 | .085 | .087 | .329 | .036 | .082 | .659 |
| Adaptive | .109 | .105 | | .302 | -.068 | .109 | .535 | .188 | .107 | .079 |
| Motor | .088 | .080 | | .273 | .228 | .084 | .007^**^ | .021 | .087 | .808 |
| Hyper *- Intercept* | .046 | .229 | | .841 | -.334 | .238 | .160 | .012 | .224 | .958 |
| *Slope* | -.114 | .088 | | .194 | -.094 | .076 | .215^ind3^ | -.117 | .080 | .145 |
| Hypo *- Intercept* | .047 | .181 | | .794 | .117 | .184 | .526 | -.279 | .175 | .110 |
| *Slope* | -.029 | .096 | | .767 | -.048 | .084 | .565 | .125 | .087 | .152 |
| SIRS *- Intercept* | -.039 | .131 | | .766 | .050 | .125 | .692 | .095 | .116 | .413 |
| *Slope* | .036 | .091 | | .692 | -.009 | .100 | .924 | -.093 | .083 | .264 |
| *Total variance explained (R^2^)* | 6.8% | | | | 16.7% | | | 21.6% | | |

1. Community participation outcomes

ind3: indirect effects via internalizing behavior (β=-.076, SE=.032, *p*=.018) and motor skills (β=-.064, SE=.031, *p*=.036). ^*^*p* < .05, ^**^*p* < .01, ^***^*p* < .001.
